# Reduction of superficial radiation dose with bolus in passive scattering proton beam therapy

**DOI:** 10.1002/acm2.13153

**Published:** 2021-01-12

**Authors:** Yeon‐Joo Kim, Chankyu Kim, Se Byeong Lee, Jae‐Sung Kim

**Affiliations:** ^1^ Proton Therapy Center National Cancer Center Korea Gyeonggi‐do Republic of Korea; ^2^ Department of Radiation Oncology Seoul National University College of Medicine Seoul National University Bundang Hospital Gyeonggi‐do Republic of Korea

**Keywords:** bolus, Monte Carlo, proton therapy, scalp, scattered protons

## Abstract

**Purpose:**

In passive scattering proton beam therapy, scattered protons from the snout and aperture increase the superficial dose, however, treatment planning systems (TPSs) based on analytic algorithms (such as proton convolution superposition) are often inaccurate in this aspect. This additional dose can cause permanent alopecia or severe radiation dermatitis. This study aimed to evaluate the effect of bolus on the superficial radiation dose in passive scattering proton beam therapy.

**Methods:**

We drew a clinical target volume (CTV) and a scalp‐p (phantom), and created plans using a TPS for a solid water phantom with and without bolus. We calculated the dose distribution in the established plans independently with Monte Carlo (MC) simulation and measured the actual dose distribution with an array of ion chambers and radiochromic films. To assess the clinical impact of bolus on scalp dose, we conducted independent dose verification using MC simulation in a clinical case.

**Results:**

In the solid water phantom without bolus, the calculated scalp‐p volume receiving 190 cGy was 20% with TPS but 80% with MC simulation when the CTV received 200 cGy. With 2 cm bolus, this decreased from 80% to 10% in MC simulation. With the measurements, average superficial dose to the scalp‐p was reduced by 5.2% when 2 cm bolus was applied. In the clinical case, the scalp‐c (clinical) volume receiving 3000 cGy decreased from 74% to 63% when 2 cm bolus was applied.

**Conclusion:**

This study revealed that bolus can reduce radiation dose at the superficial body area and alleviate toxicity in passive scattering proton beam therapy.

## 1 Introduction

It is well known that treatment planning systems (TPSs) based on analytic algorithms (such as proton convolution superposition) have limitations on dose calculations in proton beam therapy.^1^ It is also known scattered protons from the snout and aperture have dosimetric impact.^2^, ^3^ A previous study^4^ revealed that these scattered protons could increase the entrance dose in clinical practice, but commonly used TPSs do not accurately calculate this dose.

A range compensator tailors the beam, stopping distribution, and enables the dose to conform to the distal shape of the target volume.^5^ A previous study showed the dose from scattered protons can be reduced with a thicker range compensator.^4^ However, a thicker range compensator increases the risk of collision and uncertainty in dose calculation.

Bolus is often used in electron beam therapy to flatten out irregular surfaces, reduce electron penetration in parts of the field, or increase surface dose. Ideally, bolus material is equivalent to tissue in relative stopping and scattering power.^5^ We hypothesized that applying bolus on the body surface instead of a thicker range compensator could also reduce the superficial dose from scattered protons. This study aimed to evaluate the effect of bolus on the superficial radiation dose in passive scattering proton beam therapy.

## 2 Methods

### 2.A Treatment plan

**Fig. 1:**
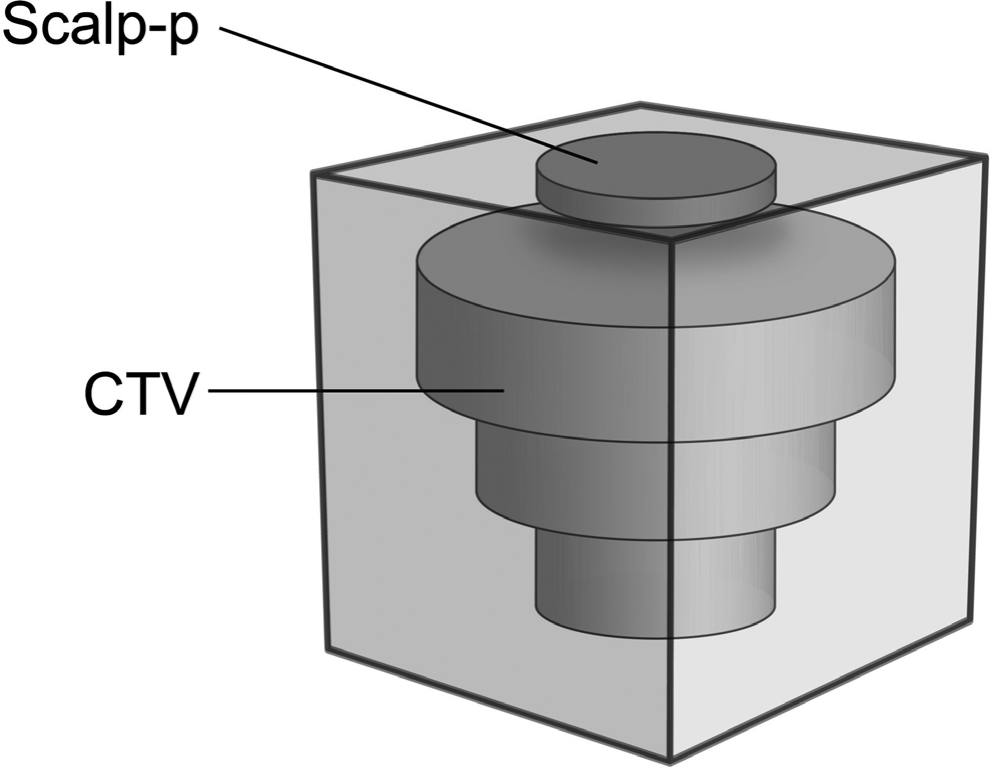
The clinical target volume (CTV) is the shape of an upside‐down three‐tiered cake with three different radii (4, 3, and 2 cm) and a 2 cm depth for each tier. This CTV was placed at a 1 cm depth from the surface. The scalp‐p (dark gray) was defined as the 2 cm radius volume above the CTV with a 5 mm depth from the surface.

We created virtual treatment plans with a TPS (Eclipse 13.7, Varian) using the proton convolution superposition algorithm^6^ based on computerized tomography (CT) images of a solid water phantom (SP34, IBA) with and without bolus (Radiation Oncology Material, Republic of Korea). The relative proton stopping powers were measured using a multilayer ionization chamber (Zebra, IBA) as 1.03 and 0.95 for the solid water phantom and bolus, respectively. We drew a clinical target volume (CTV) in the shape of an upside‐down three‐tiered cake with 4, 3, and 2 cm radii and 2 cm depth for each tier. This CTV was placed at a depth of 1 cm from the surface. We defined a superficial volume 2 cm in radius and 5 mm in depth as the “scalp‐p (phantom)” (Figure 1). The aperture and range compensator were designed to cover the CTV taking into consideration the snout size (10 cm diameter), milling compensation (1.2 cm drill bit), and minimum thickness of the range compensator (2 mm). The area corresponding to the scalp‐p in the range compensator was the thinnest area. The distance from the end of the range compensator to the top of the solid water phantom was fixed at 8.5 cm. The plans were normalized to deliver 200 cGy to a minimum of 95% of the CTV. The range of the proton beam was adjusted to cover the CTV. The plan was evaluated using a dose distribution and a dose‐volume histogram (DVH). The DVH is a plot of the volume (Y‐axis, %) of a given structure receiving a certain dose or higher, as a function of dose (X‐axis, cGy).^5^

### 2.B Dose verification based on Monte Carlo (MC) simulation

MC simulations generally exhibit more accurate dose calculations than TPSs in proton therapy because they consider all the components in the beam pathway, such as the range modulator, collimators, snout, aperture, and range compensator, whereas TPSs consider them in a limited way. In previous studies,^7^, ^8^ a MC simulation system based on a particle simulation tool (TOPAS)^9^ for passive scattering proton therapy was developed and validated for independent dose verification of treatment plans. In this study, we used that MC simulation system to conducted MC simulation for each treatment plan, and the results were imported into the TPS for comparison and analysis. The imported dose was normalized to the dose at the middle of the spread‐out Bragg peak.

### 2.C Measurement of superficial dose

To verify the effect of bolus on the superficial dose, we measured the dose distribution in the solid water phantom with an array of ion chambers (MatriXX, IBA) and radiochromic films (EBT3, GaFchromic) using the IBA proton therapy machine at our center. The used MatriXX was calibrated using a farmer‐type ionization chamber (Farmer chamber model 30013, PTW Freiburg) considering relative biological effectiveness of 1.1 for protons. The measurement was performed at 4 mm depth in consideration of the equivalent thickness of the MatriXX and dimensions of the scalp‐p. The aperture and range compensator, composed of brass and poly methyl methacrylate (PMMA), respectively, were manufactured as indicated in the plan and mounted to the snout. The irradiated films were scanned and radiation doses were determined using the RIT software (Radiological Imaging Technology, Inc., Colorado Springs, CO, USA) with calibration curves previously determined using a farmer‐type ionization chamber (Farmer chamber model 30013, PTW Freiburg).

The TPS and MC calculated 2D dose distributions were imported to the MatriXX operating software (OmniPro‐ImRT, IBA). The TPS and MC calculated and measured dose distributions were interpolated using 1 mm spacing and aligned to match the center. The converted dose distributions were compared in orthogonal profiles and 2D gamma index analysis.

Gamma index analysis is widely adopted for patient‐specific quality assurance to evaluate agreement between calculated and measured dose distributions by utilizing the percent dose difference and distance to agreement (DTA).^10^ In this study, we conducted gamma index analysis to evaluate the difference in doses measured using MatriXX with and without bolus. For this purpose, the DTA was fixed at a minimum (1 mm), and the gamma index analysis was repeated with varying dose difference criteria.

### 2.D Simulation in a clinical case

To assess the clinical impact of bolus on scalp dose, we performed MC simulation in a clinical patient who had been treated at our center without bolus. The patient was a 37‐year‐old female with WHO grade III, MGMT methylation (+) anaplastic astrocytoma at the right frontal lobe. She underwent subtotal tumor removal and received postoperative proton beam therapy (6000 cGy in 25 fractions) with temozolomide. We drew a “scalp‐c (clinical)” as a 5 mm deep superficial volume where the proton beam entered. For the Hounsfield unit (HU)‐assigned bolus contour in the MC simulation, we created virtual CT images from the original CT images using in‐house software. The software recognized bolus contour and filled the inside of the contour with the assigned HU value. Finally, parts of the original CT images were overwritten with these filled contour images. The MC simulation was conducted using these virtual CT images. Normalization in the MC simulation was adjusted so that 100% of the prescribed dose covered 95% of the CTV.

## 3 Results

### 3.A MC simulation

#### 3.A.1 Difference between TPS and MC calculated dose

**Fig. 2:**
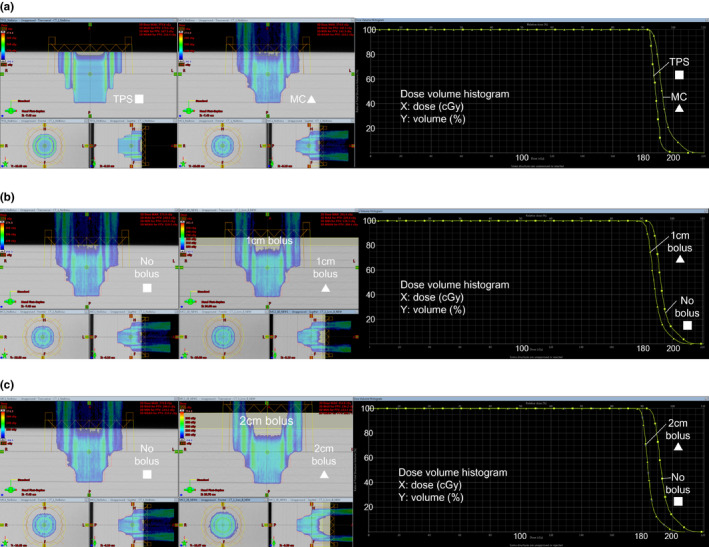
(a) Without bolus, the calculated scalp‐p volume receiving 190 cGy was 20% with the treatment planning system (TPS) and 80% with the Monte Carlo (MC) simulation. The dose distribution is presented on the left and dose‐volume histogram (DVH) on the right. (b) With 1 cm bolus, the scalp‐p volume receiving 190 cGy decreased from 80% to 30%. The dose distribution is presented on the left and DVH on the right. (c) With 2 cm bolus, the scalp‐p volume receiving 190 cGy decreased from 80% to 10%. The dose distribution is presented on the left and DVH on the right.

We compared the TPS and MC calculated scalp‐p dose for the plan without bolus. The calculated scalp‐p volume receiving 190 cGy was 20% with TPS but 80% with MC simulation [Figure 2a]. The differences between the TPS and MC calculated dose received by 50% and 5% of the scalp‐p volume (D50% and D5%) were 5 and 15 cGy, respectively. These dose differences are thought to be due to the limitations of the TPS analytical algorithm. In particular, the additional dose due to scattered protons from the snout and aperture is not included in the TPS calculation.

#### 3.A.2 MC simulation of bolus effect

To demonstrate the effect of bolus on superficial dose, we compared MC simulation results from plans with and without bolus. The scalp‐p volume receiving 190 cGy decreased from 80% without bolus to 30% with 1 cm bolus [Figure 2b]. The differences between no bolus and 1 cm bolus D50% and D5% were both 5 cGy. When bolus thickness was increased from 1 to 2 cm, the scalp‐p dose reduction was more prominent. The scalp‐p volume receiving 190 cGy was 10% with 2 cm bolus [Figure 2c]. The differences between no bolus and 2 cm bolus D50% and D5% were both 10 cGy. These results indicate that bolus reduced the scalp‐p dose and this effect was greater with 2 cm bolus than 1 cm.

#### 3.A.3 Comparison of TPS and MC calculations with bolus

With 2 cm bolus, the effect of scattered protons was reduced, which also reduced the difference between the TPS and MC calculations (Supplementary Figure S1). The remaining difference was thought to be due to the limitations of the TPS calculation, mainly the differences in the calculation of the scattering at the edge of the compensator.

### 3.B Verification with measurements

#### 3.B.1 Comparison of TPS and MC calculations and measurements

**Fig. 3:**
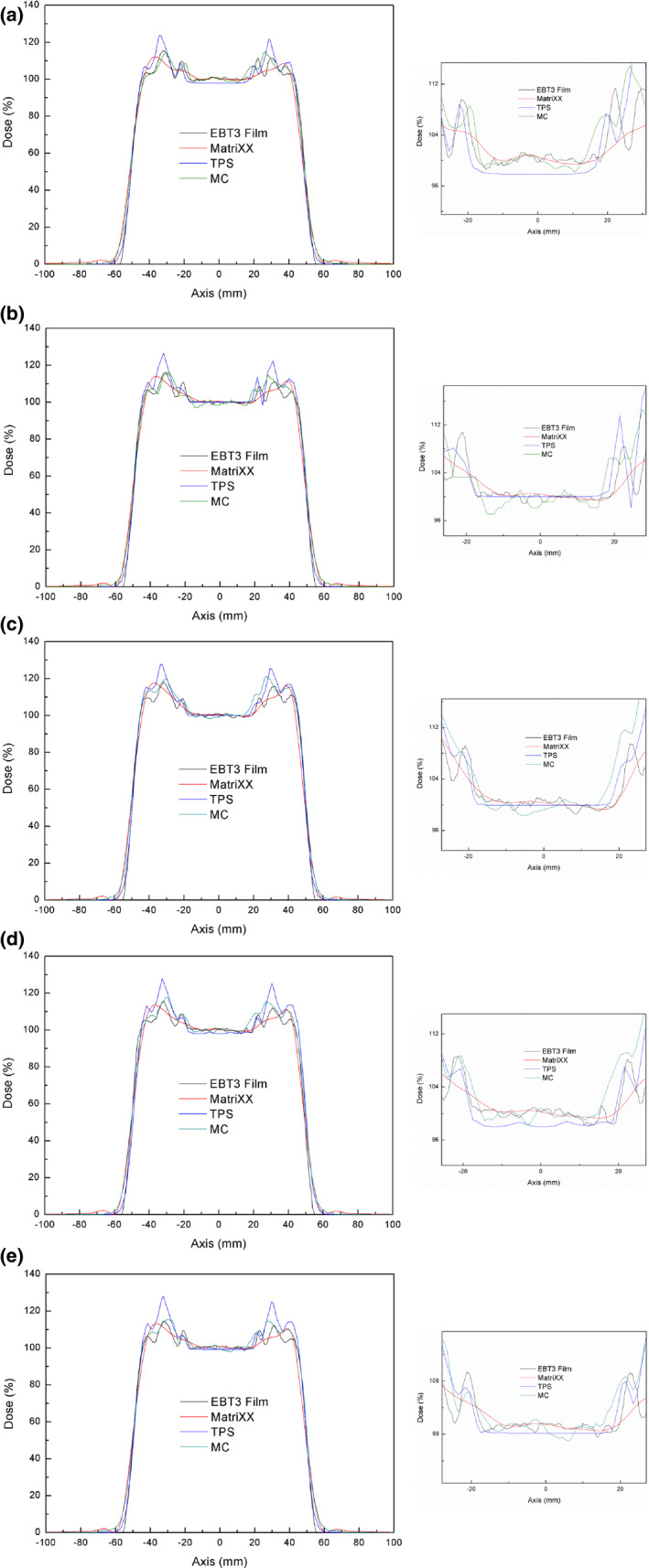
The treatment planning system (TPS) vs. Monte Carlo (MC) simulation vs. MatriXX vs. film scalp‐p doses with (a) no bolus, (b) 1 cm bolus, (c) 2 cm bolus, (d) 1 cm bolus with 1 cm air gap, and (e) 1 cm bolus with 2 cm air gap.

To verify the reduction in superficial dose due to bolus, we compared TPS and MC calculated dose distributions with those measured using MatriXX and films. Figure 3 presents the TPS vs MC vs MatriXX vs film scalp‐p doses for the no bolus, 1 cm bolus, 2 cm bolus, 1 cm bolus with 1 cm air gap, and 1 cm bolus with 2 cm air gap treatment plans. When the lower spatial resolution (7 mm) of MatriXX was considered, the doses from the MC simulation and those measured using MatriXX and film were consistent with each other for all plans.

However, the TPS calculated scalp‐p dose without bolus was smaller than the others by about 2.7% [Figure 3a]. This was because the scattered protons from the snout and aperture were not included in the TPS analytical algorithm. With an air gap, the TPS calculated scalp‐p dose was also smaller than the others [Figure 3d,e]. This was considered to be due to the limitation in the TPS dose calculation with an air gap.

#### 3.B.2 2D analysis of measured dose distribution

**Fig. 4:**
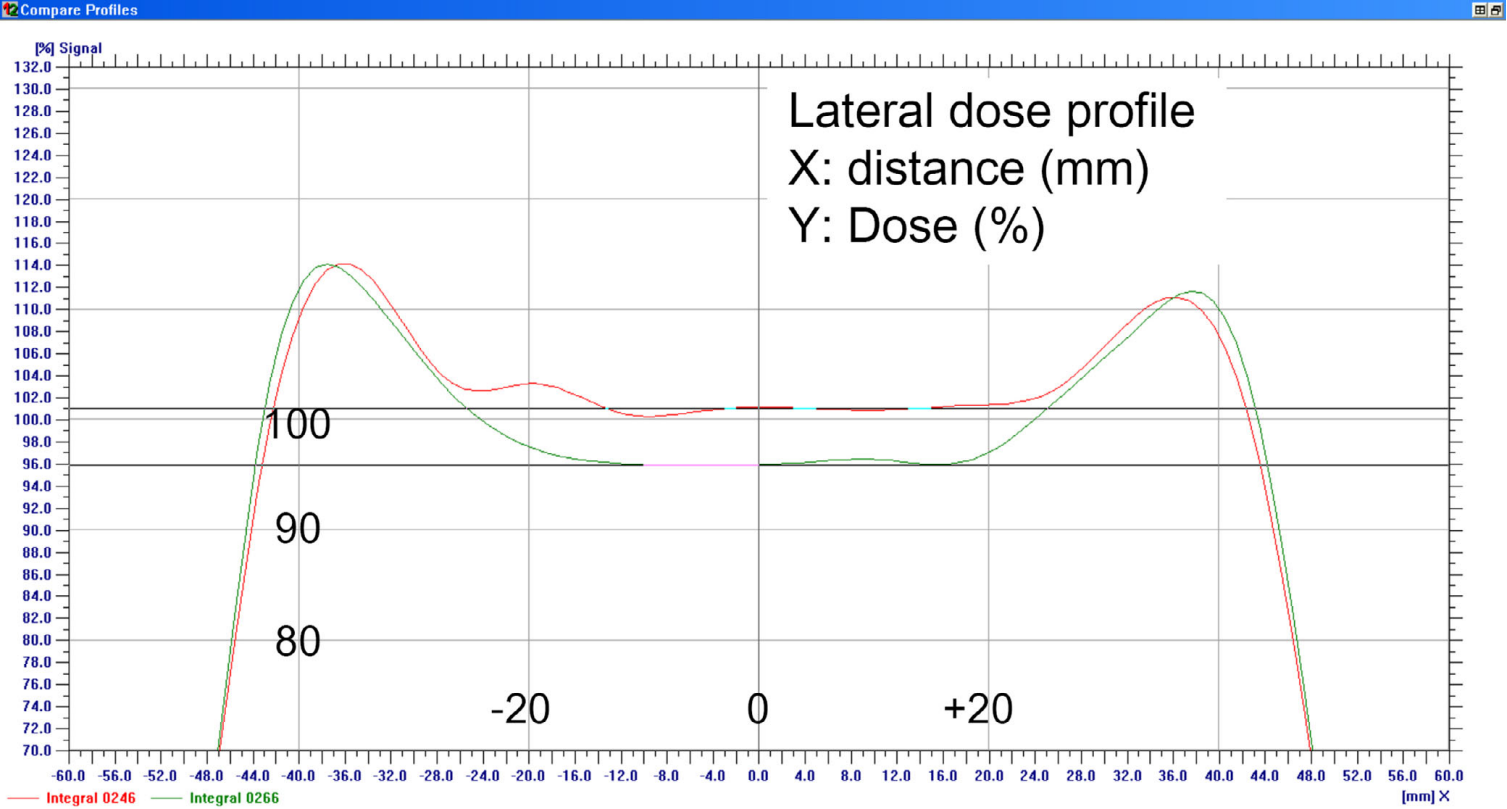
The relative dose difference between no bolus (red) and 2 cm bolus (green) in the region of interest (ROI) was 5.2% in the lateral dose profile.

We conducted a 2D gamma index analysis using MatriXX to evaluate how bolus affected the measured scalp‐p dose. For this purpose, the DTA was fixed at a minimum (1 mm), and the region of interest (ROI) was set at the central 4 × 4 cm area to exclude the difference in the lateral penumbra. Table 1 presents the MatriXX measurement accordance rates on gamma analysis for various criteria; (1) 3% and 1 mm, (2) 2% and 1 mm, and (3) 1% and 1 mm. Accordance between scalp‐p dose with no bolus and 2 cm bolus was only 20.15%, even with 3% and 1 mm criteria, meaning the dose difference in the ROI exceeded 3%. In the beam profile, the average dose in the ROI was reduced by 5.2% with 2 cm bolus [Figure 4]. The dose difference in the ROI between no bolus and 1 cm bolus was about 2.6%, and the accordance rate, depending on the dose criteria (3% and 2%), accurately reflected this dose difference.

**Table 1:** MatriXX measurement accordance rate (%) on gamma analysis.

	Criteria		
	3% and 1 mm	2% and 1 mm	1% and 1 mm
No bolus vs 1 cm bolus	85.70	33.64	0.00
No bolus vs 2 cm bolus	20.15	10.80	0.00
1 cm bolus vs 1 cm bolus with 1 cm air gap	100.00	100.00	100.00
1 cm bolus vs 1 cm bolus with 2 cm air gap	100.00	100.00	97.62

### 3.C Effect of an air gap

#### 3.C.1 Difference between TPS and MC calculated dose

When applying bolus to an irregular surface, such the ear, there can be an air gap between bolus and body surface. Therefore, we simulated this condition with our bolus and solid water phantom. With a 1 cm air gap between bolus and solid water phantom, the calculated scalp‐p volume receiving 190 cGy was 0% with TPS and 30% with MC simulation [Supplementary Figure S2a]. The differences between the TPS and MC calculated D50% and D5% were 5 and 10 cGy, respectively. This might be due to inaccurate TPS dose calculation caused by the air gap between bolus and solid water phantom.

In the MC simulation, dose reduction by bolus was not compromised by the air gap. There was no difference in the scalp‐p dose with or without the air gap in the MC simulation [Supplementary Figure S2b].

#### 3.C.2 Verification with measured results

To verify the measurements, the measured dose in the plans with and without bolus was compared. Even with a 3% and 1 mm threshold, the accordance rate on gamma analysis was almost 100% between 1 cm bolus vs 1 cm bolus with an air gap (Table 1). Thus, we can assume that the air gap did not affect the scalp‐p dose.

### 3.D Simulation in a clinical case

#### 3.D.1 Difference between the TPS and MC calculated dose

**Fig. 5:**
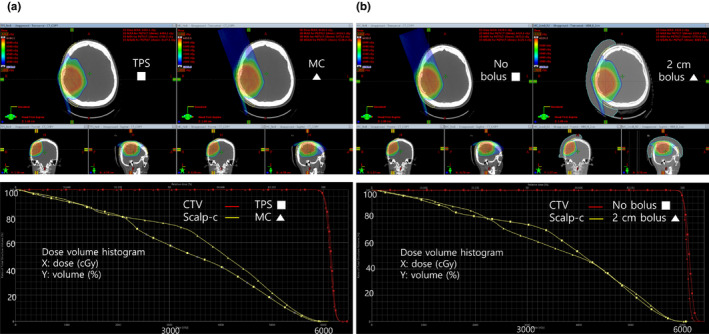
(a) Without bolus, the calculated scalp‐c volume receiving 3000 cGy was 58% with the treatment planning system (TPS) and 74% with the Monte Carlo (MC) simulation. The dose distribution is presented on the top and dose‐volume histogram (DVH) on the bottom. (b) With 2 cm bolus, the scalp‐c volume receiving 3000 cGy decreased from 74% to 63%. The dose distribution is presented on the top and DVH on the bottom.

The dose distribution and DVH with no bolus are presented in Figure 5a. The calculated scalp‐c volume receiving 3000 cGy was 58% with TPS and 74% with MC simulation. This finding is consistent with the previously mentioned limitations of the TPS calculation.

#### 3.D.2 MC simulation of bolus effect

The dose distribution and the DVH with 2 cm bolus are presented in Figure 5b. The scalp‐c volume receiving 3000 cGy decreased from 74% to 63%. With 1 cm bolus, the scalp‐c volume receiving 3000 cGy was 65% [Supplementary Figure S3a]. The scalp‐c dose decreased when bolus thickness was increased from 1 to 2 cm [Supplementary Figure S3b]. These findings are in line with those from the solid water phantom.

## 4 Discussion

The present study was designed to determine whether bolus applied on the body surface could reduce the superficial radiation dose from scattered protons in passive scattering proton beam therapy. In our simulation in a clinical case, 2 cm bolus reduced the scalp‐c volume receiving 3000 cGy by 11%. Considering that permanent alopecia may be correlated with dose exposure of 3000 cGy in combination with conventional chemotherapy,^11^ bolus can reduce permanent alopecia in patients receiving passive scattering proton beam therapy. To our knowledge, this is a novel finding with great clinical impact.

A previous study evaluated the use of a patient‐specific bolus to replace multiple field‐specific range compensators during passive scattering proton delivery.^12^ It showed dosimetric equivalency between the range compensator and bolus plans. Another study found a patient‐specific bolus reduced the mean dose to all organs at risk compared with the snout and nozzle‐mounted range shifter in intensity‐modulated proton therapy of superficial lesions.^13^ However, neither study evaluated the superficial dose with bolus.

When the thickness of a range compensator increased, the range of proton beam also increased to cover the same target volume. When a proton beam of higher energy is used, the directional nature of the proton beam is intensified in the forward direction which can lead to reduced scattering at the snout and aperture.^4^ Applying of bolus could also reproduce this phenomenon. When we put bolus on the body surface, the range of proton beam needed to be increased to cover the target, thus reducing the scattered protons.

Dose reduction was found to be dependent on range compensator thickness in a previous study which compared a 2‐mm‐base range compensator with 2‐cm‐base and 4‐cm‐base range compensators.^4^ In our study, the scalp‐p dose reduction was more substantial with 2 cm bolus than 1 cm. Future studies should evaluate if there is a ceiling to this effect. However, in practice, it is not easy to apply bolus thicker than 2 cm to the patient due to weight of bolus.

In this study, we found that an air gap between bolus and body surface did not affect the superficial dose in the MC simulation, MatriXX, and film, whereas it did in the TPS. Since radiation oncologists evaluate plans in a TPS, it is desirable to reduce the air gap between bolus and the body surface as much as possible. For an irregular body surface, a patient‐specific bolus could be made using a 3D printer or moldable bolus. In the current study, we demonstrated that MC simulation is consistent with measured results obtained from MatriXX or film. Therefore, if the proton water equivalent thickness value of a bolus material is known, the dose reduction effect of any kind of bolus can be simulated.

This study has several limitations. First, we could not compare passive scattering to pencil beam scanning (PBS) because MC simulation was only available for passive scattering in our center. However, we observe an excellent dose distribution agreement at the superficial region between TPS calculations and quality assurance measurements for PBS plans. Scattered protons rarely exist in our PBS beam mode because the proton beam is not scattered by nozzle components. If a multileaf collimator or aperture is applied to reduce lateral penumbra in PBS, scattered protons should be considered. Second, we performed MC simulation in only one clinical case. In a future study, we will evaluate more clinical cases and compare passive scattering to PBS with regard to superficial dose in individual patients.

## 5 Conclusion

This study revealed that bolus can reduce the superficial radiation dose and alleviate toxicity in passive scattering proton beam therapy.

## Conflict of interest

No conflict of interest.

## Supporting information


**Fig S1**. With 2 cm bolus, the dose difference between the treatment planning system (TPS) and Monte Carlo (MC) simulation decreased. The dose distribution is presented on the left and dose‐volume histogram (DVH) on the right.

**Fig S2A**. With a 1 cm air gap between bolus and solid water phantom, the calculated scalp‐p volume receiving 190 cGy was 0% with the treatment planning system (TPS) and 30% with the Monte Carlo (MC) simulation. The dose distribution is presented on the left and dose‐volume histogram (DVH) on the right.

**Fig S2B**. In the MC simulation, dose reduction by bolus was not compromised by an air gap. There was no difference in the scalp‐p dose with or without the air gap in the MC simulation. The dose distribution is presented on the left and DVH on the right.

**Fig S3A**. With 1 cm bolus, the scalp‐c volume receiving 3000 cGy decreased from 74% to 65%. The dose distribution is presented on the top and dose‐volume histogram (DVH) on the bottom.

**Fig S3B**. The scalp‐c dose decreased when bolus thickness increased from 1 to 2 cm.
