# Supplementary figures and images for: Reduction of superficial radiation dose with bolus in passive scattering proton beam therapy
Source: J Appl Clin Med Phys. 2021 Jan 12;22(2):69–76. doi: 10.1002/acm2.13153 (PMC7882114; doi:10.1002/acm2.13153)

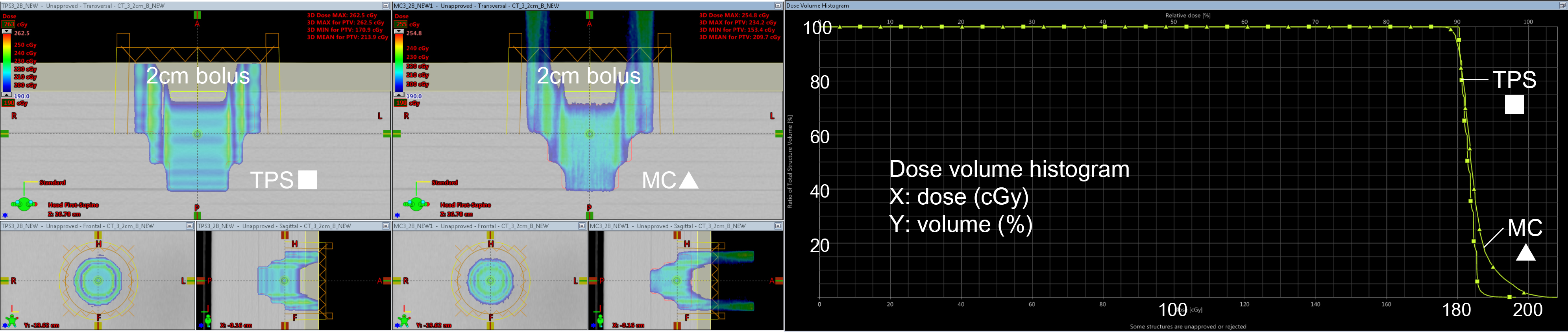

Supplement: Supplementary file 1 — Fig S1. With 2 cm bolus, the dose difference between the treatment planning system (TPS) and Monte Carlo (MC) simulation decreased. The dose distribution is presented on the left and dose‐volume histogram (DVH) on the right. [file ACM2-22-69-s001.jpg]

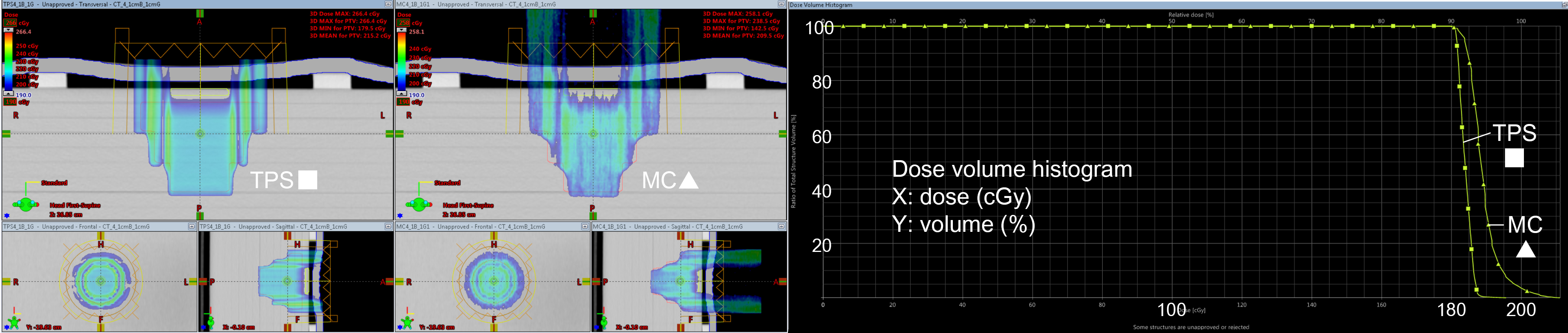

Supplement: Supplementary file 2 — Fig S2A. With a 1 cm air gap between bolus and solid water phantom, the calculated scalp‐p volume receiving 190 cGy was 0% with the treatment planning system (TPS) and 30% with the Monte Carlo (MC) simulation. The dose distribution is presented on the left and dose‐volume histogram (DVH) on the right. [file ACM2-22-69-s002.jpg]

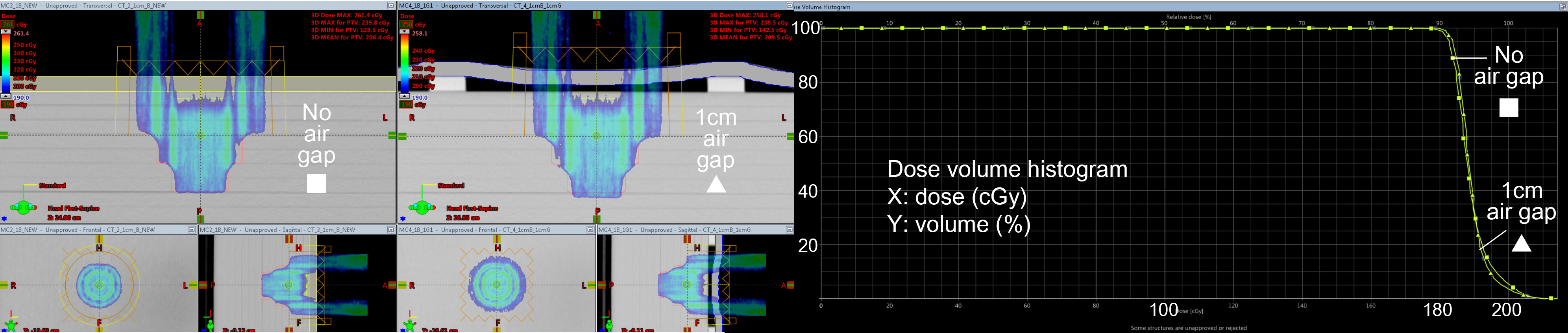

Supplement: Supplementary file 3 — Fig S2B. In the MC simulation, dose reduction by bolus was not compromised by an air gap. There was no difference in the scalp‐p dose with or without the air gap in the MC simulation. The dose distribution is presented on the left and DVH on the right. [file ACM2-22-69-s003.jpg]

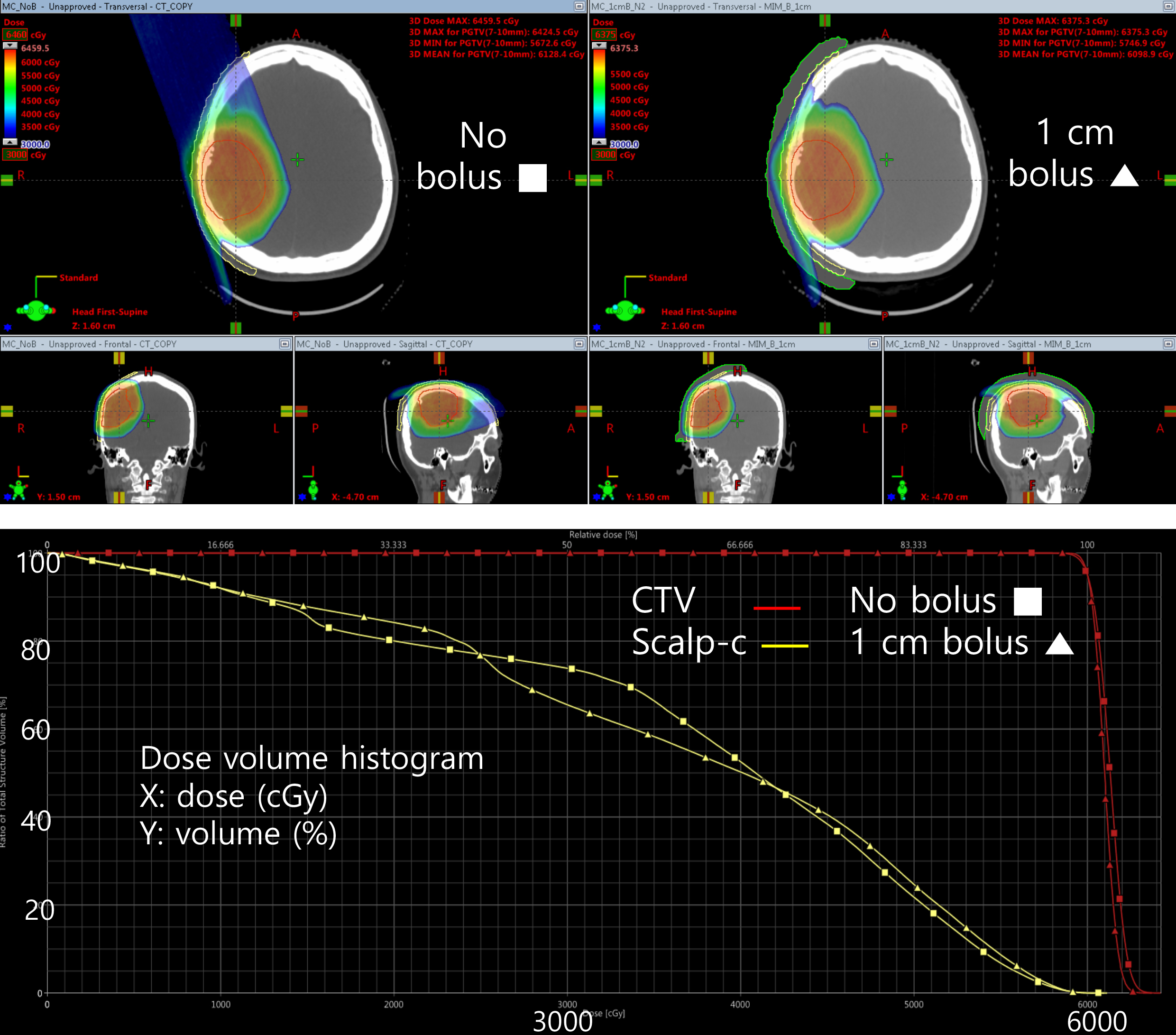

Supplement: Supplementary file 4 — Fig S3A. With 1 cm bolus, the scalp‐c volume receiving 3000 cGy decreased from 74% to 65%. The dose distribution is presented on the top and dose‐volume histogram (DVH) on the bottom. [file ACM2-22-69-s004.jpg]

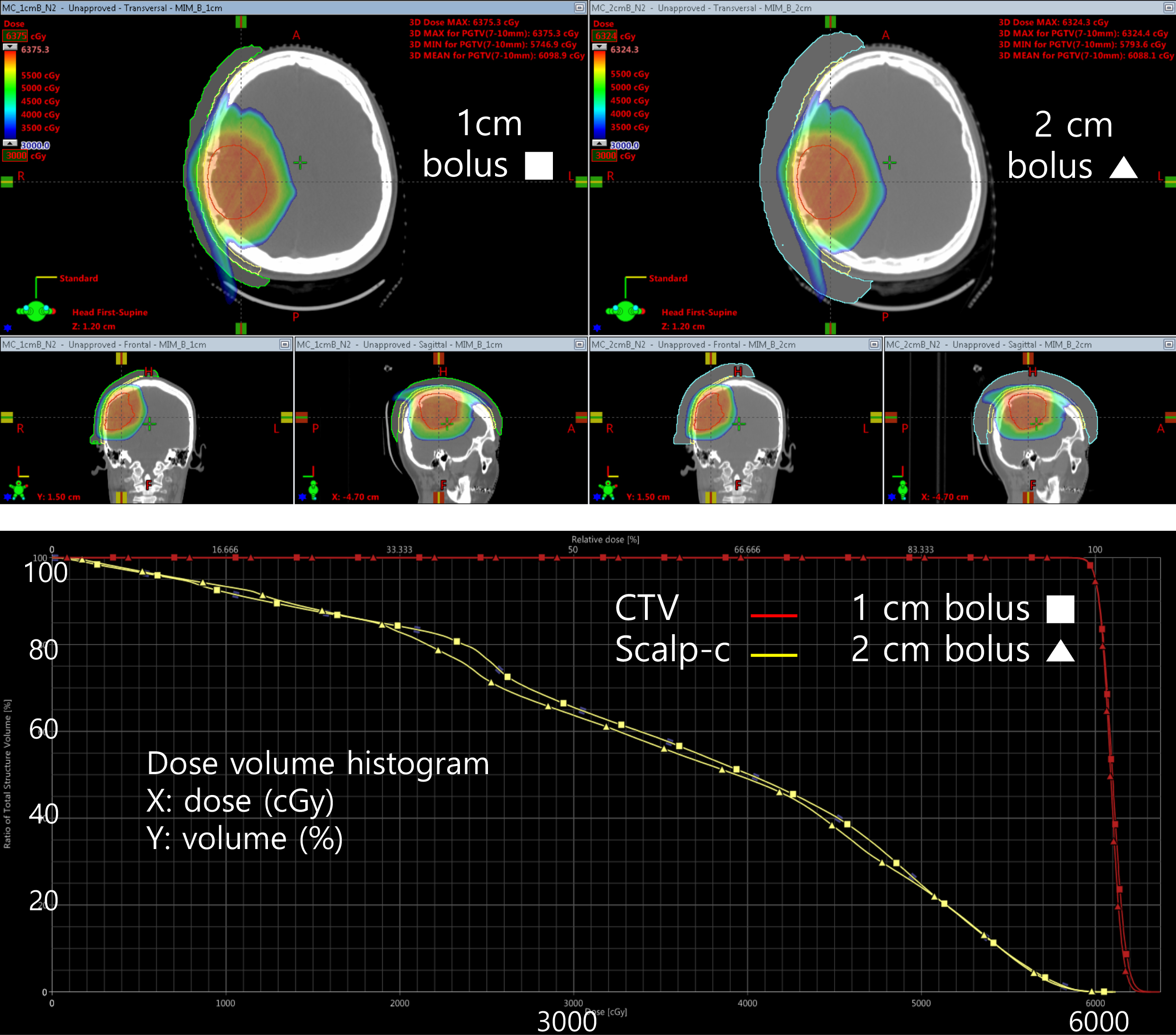

Supplement: Supplementary file 5 — Fig S3B. The scalp‐c dose decreased when bolus thickness increased from 1 to 2 cm. [file ACM2-22-69-s005.jpg]
